# Differential conformational modulations of MreB folding upon interactions with GroEL/ES and TRiC chaperonin components

**DOI:** 10.1038/srep28386

**Published:** 2016-06-22

**Authors:** Satish Babu Moparthi, Uno Carlsson, Renaud Vincentelli, Bengt-Harald Jonsson, Per Hammarström, Jérôme Wenger

**Affiliations:** 1 CNRS, Aix Marseille Université, Centrale Marseille, Institut Fresnel, 13013 Marseille, France; 2 IFM, Department of Chemistry, Linköping University, 581 83 Linköping, Sweden; 3 Architecture et Fonction des Macromolécules Biologiques (A.F.M.B), UMR7257 CNRS, Université Aix-Marseille, Case 932, 163 Avenue de Luminy, 13288 Marseille Cedex 9, France

## Abstract

Here, we study and compare the mechanisms of action of the GroEL/GroES and the TRiC chaperonin systems on MreB client protein variants extracted from *E. coli*. MreB is a homologue to actin in prokaryotes. Single-molecule fluorescence correlation spectroscopy (FCS) and time-resolved fluorescence polarization anisotropy report the binding interaction of folding MreB with GroEL, GroES and TRiC. Fluorescence resonance energy transfer (FRET) measurements on MreB variants quantified molecular distance changes occurring during conformational rearrangements within folding MreB bound to chaperonins. We observed that the MreB structure is rearranged by a binding-induced expansion mechanism in TRiC, GroEL and GroES. These results are quantitatively comparable to the structural rearrangements found during the interaction of β-actin with GroEL and TRiC, indicating that the mechanism of chaperonins is conserved during evolution. The chaperonin-bound MreB is also significantly compacted after addition of AMP-PNP for both the GroEL/ES and TRiC systems. Most importantly, our results showed that GroES may act as an unfoldase by inducing a dramatic initial expansion of MreB (even more than for GroEL) implicating a role for MreB folding, allowing us to suggest a delivery mechanism for GroES to GroEL in prokaryotes.

The chaperonins are large, multimeric, barrel-shaped proteins, which assist in the folding and prevent aggregation of non-native proteins. The chaperonin family is ubiquitous in all forms of life and can be divided into two main classes:  chaperonins of group I are found in eubacteria and organelles with prokaryotic origin, while chaperonins of group II are present in archaea and in the cytoplasm of eukaryotes. Both group I and group II chaperonins are large oligomeric complexes, and share a similar general architecture composed of two identical 7–9-membered rings stacked back to back in a doughnut shape. Each ring contains a central cavity modulating non-native polypeptides (substrate proteins) during folding^1^,^2^,^3^,^4^,^5^,^6^,^7^,^8^,^9^,^10^,^11^. Among the group I chaperonins, the GroEL/GroES complex in *E. coli* has received a considerable interest. GroEL is a double-ring 14mer with a hydrophobic patch at its opening, while GroES is a single-ring heptamer that binds to GroEL in the presence of ATP, acting like a lid to cover the GroEL folding cavity. The equivalent to GroEL/GroES complex in eukaryotes is the tail-less complex polypeptide 1 ring complex TRiC chaperonin (TCP-1 Ring Complex, also called CCT for chaperonin containing TCP-1). Although the TRiC and GroEL/ES systems share many similarities and have received a lot of attention, there are still fundamental open questions and controversies about their mechanisms of action and substrate recognition^12^.

The mere binding of the target protein to GroEL has been shown to induce modulation in the chaperonin, and lead to the conformational rearrangement of the target protein^2^,^13^,^14^,^15^,^16^. To complete this GroEL binding-induced expansion, the addition of GroES and ATP induces a further compaction of the bound target protein, and improves its refolding into the active conformation^14^,^17^,^18^,^19^,^20^. On the other hand, the eukaryotic chaperonin TRiC has been shown to recognize and bind the target proteins more specifically than its bacterial counterpart. For instance, we have demonstrated that β-actin is actively stretched by binding-induced expansion by TRiC^21^, while the GroE chaperonin is not able to guide actin into its native state due to incomplete binding induced unfolding (stretching) of the central ATP binding site^13^. To explain this difference, it has been proposed that the two different chaperonins recognize different folding intermediates of the actin protein^22^. In the meanwhile, many substrate proteins were discovered for TRiC^23^, yet β-actin remains the main substrate used in most TRiC studies due to its significant role in the maintenance of cell shape^24^.

In this report, we study and compare the mechanisms of action of the GroE and TRiC systems with MreB client protein variants extracted from *E. coli*. MreB is a homologue of actin found in prokaryotes, where it plays a crucial role in controlling several important physiological processes such as cell shape, division or locomotion. The crystal structure of MreB from *Thermotoga maritima* revealed that MreB is very similar to the three dimensional structure of actin although they share only 15% the amino acid sequence homology^25^. MreB is an obligate or stringent natural substrate for the GroE chaperonin^26^,^27^, and as a homologue of actin, MreB is also an interesting substrate to investigate the evolutionary aspects found in TRiC interaction. We recently showed that both GroEL and GroES either separately or working together remodulated MreB during folding including binding induced expansion and compression^28^.

From observing similarities and differences in the action of the chaperonins, we identify conformational rearrangements in MreB, which are specific for each chaperonin. We use single molecule setups for fluorescence correlation spectroscopy (FCS) and time-resolved fluorescence anisotropy to study the interactions between MreB protein variants and the GroE or TRiC systems. Additionally, homo-FRET measurements indicate that the MreB structure is rearranged by a binding-induced expansion mechanism in TRiC, GroEL and GroES. Moreover, the binding to chaperonins causes the separation of the two subdomains, leading to an expansion of the ATP-binding cleft in MreB. The pronounced compaction of TRiC-bound MreB, after addition of AMP-PNP to the chaperonin complex, is as dramatic as when AMP-PNP and GroES are added to the MreB−GroEL complex. Most importantly we demonstrate that GroES acts as a superior unfoldase compared to GroEL and TriC by inducing a significant expansion of MreB which may be crucial for efficient MreB folding *in vivo*.

## Results and Discussion

**Figure 1:**
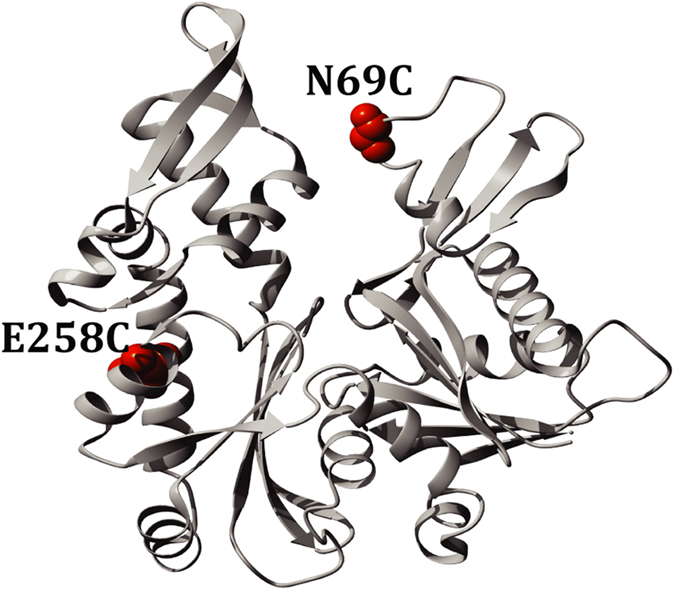
Structure of native MreB. The corresponding site-specific labeling positions were selected by sequence alignment in which *E. coli* MreB was modeled on top of the crystal structure of MreB *Thermatoga maritima* [Protein Data Bank (PDB) accession number 1JCE] in YASARA v9.11.23.

In the present study, we introduced cysteines at two positions in a cysteine-free pseudo-wild-type MreB in which the natural cysteine residues have been substituted by alanine to produce the three MreB variants, N69CMreB, E258CMreB, and N69CE258CMreB (Fig. 1).

### MreB - Chaperonin complexes probed by FCS

**Figure 2:**
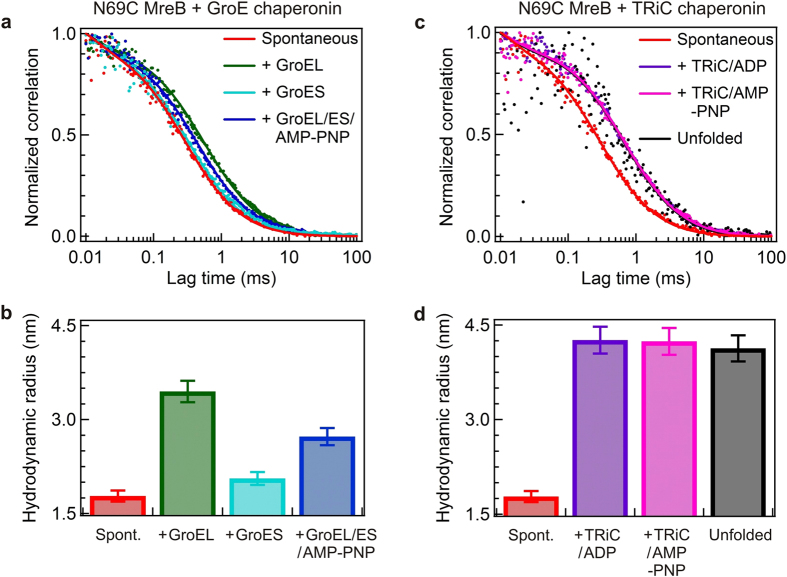
FCS traces and apparent hydrodynamic radius of N69CMreB with various chaperonins. Normalized FCS correlation traces (**a**) and apparent hydrodynamic radius (**b**) of the Atto647 labelled N69CMreB variant in the combination of GroE chaperonin components. Normalized FCS correlation traces (**c**) and corresponding apparent hydrodynamic radius (**d**) of the Atto647 labelled N69CMreB variant in the presence of the TRiC chaperonin. The chaperonin components are present from the beginning before addition of the denatured N69CMreB to refolding buffer in the presence of GroEL alone (*green*), GroES alone (*cyan*), GroEL/ES/AMP-PNP (*blue*), TRiC/ADP (*maroon*), TRiC/AMP-PNP (*pink*) and in comparison with the spontaneous refolding (*red*). In all experiments the concentrations of GroEL, TRiC and GroES were 250, 250 and 350 nM, respectively, corresponding to a 1.6-fold molar excess of GroEL, TRiC and 2.2-fold molar excess of GroES relative to the concentration of MreB.

FCS should provide an ideal method for examining the interaction between MreB and GroEL alone, GroES alone, in concert, or the TRiC chaperonine system captured during the refolding process (Fig. 2). By employing a minute confocal volume and a low protein concentration (single-molecule) setup this technique allows discrimination of single protein complexes and readily avoids monitoring of protein aggregates, which may interfere in bulk measurements. The measured translational diffusion time by FCS depends on the size and shape of the MreB-chaperonin complex, which is much longer than that of the unbound MreB monomer. When a non-native MreB intermediate binds with GroEL alone, we observe a substantial shift in the FCS curve of the spontaneous refolding of the MreB monomer (Fig. 2a). Interestingly, TRiC mediated non-native MreB folding also shows a significant increase in translational diffusion time when compared to spontaneous refolding (Fig. 2c). These data clearly demonstrate the binding between TRiC and MreB.

Several studies have shown that ATP binding and hydrolysis result in conformational rearrangement in the substrate recognizing apical domains of the chaperonins, which results in the modulation and release of the substrate proteins^4^. However, native-like MreB is prone to aggregate as it is released from the chaperonin cavity in the refolding buffer. To avoid this effect, we use AMP-PNP as a non-hydrolyzable ATP analogue. Even with addition of AMP-PNP to the GroEL/ES and TRiC chaperonins, the FCS curves reveal similar translational diffusion correlation times like the MreB/GroEL or MreB/TRiC/ADP complexes, respectively, indicative of a productive Mreb-GroEL/ES/AMP-PNP complex or MreB/TRiC/AMP-PNP complex and not release of the committed MreB into free solution during folding (Fig. 2a,c). Because the translational diffusion of MreB is dependent upon the size of the molecule, the release of an MreB monomer with a molecular weight of about ~37 kDa would cause a significant drop in diffusion time compared to when it is bound to the 800–900 kDa complexes of the chaperonins.

In all cases direct fitting of the observed FCS data into one-species fit (Fig. 2a,c) allowed us to assume that the entire MreB molten globule intermediate population was captured by the GroE or TRiC chaperonins at the beginning of the refolding reaction. Notably unfolded MreB (in 4 M GuHCl) showed a large apparent hydrodynamic radius (>4 nm). The apparent hydrodynamic radius deduced from the FCS data in Fig. 2a,c are summarized in Fig. 2b,d respectively for the N69CMreB variant. In GroEL alone mediated refolding the apparent hydrodynamic radius of N69CMreB increased from 1.8 to 3.4 nm while the GroES alone mediated refolding did not noticeably affect the apparent hydrodynamic radius (Fig. 2b). Furthermore, successive additions of GroES and AMP-PNP to the N69CMreB/GroEL complex do modify the apparent hydrodynamic radius of N69CMreB from 3.4 to 2.7 nm (Fig. 2b). This result indicates that GroES and AMP-PNP addition is further able to modulate the N69CMreB mutant in the presence of GroEL. Unlike the GroEL mediated refolding, the FCS traces obtained in TRiC/ADP mediated refolding showed a similar diffusion dynamics even in the presence of AMP-PNP nucelotide with an apparent hydrodynamic radius of 4.2 nm (Fig. 2d). This indicates that the N69CMreB apparent hydrodynamic radius increased ~2.4 nm in the presence of TRiC when compared with spontaneous refolding, but does not significantly change with the subsequent addition of non-hydrolysable AMP-PNP nucleotide to the complex.

We also obtained similar results for the other single mutant E258CMreB and the double mutant N69CE258CMreB in the presence of both GroE and TRiC chaperonin assisted refolding when compared to spontaneous refolding (Supplementary Fig. S1). All MreB mutants share a comparable apparent hydrodynamic radius of 1.8 nm at pH 7.5.

The calculated apparent hydrodynamic radius enables us to draw several conclusions: (1) non-native MreB is able to form complex with GroEL alone, and TRiC alone; (2) committed MreB molecules are not released from MreB-GroEL/ES/AMP-PNP or MreB/TRiC/AMP-PNP complexes due to the lack of ATP hydrolysis during folding (3) due to the small size of the MreB-GroES complex, FCS only detected a minor change from 1.7 nm (spontaneous) to 2.0 nm apparent hydrodynamic radius increase for MreB in complex with GroES. It has been proven that changes in the diffusion coefficient that is required to resolve distinct binding has to be larger than 1.6, which corresponds to a (1.6)^3^ = 4-fold minimum increase in the molecular weight^29^. However, the difference in molar mass of the 110-kDa MreB-GroES complex compared to the non-native 37-kDa MreB monomer itself is not sufficient to induce a significant change in apparent hydrodynamic radius as observed by FCS. Hence, it is not possible to reliably observe the formation of the MreB/GroES complex using only diffusion-based FCS analysis.

### Time resolved fluorescence anisotropy confirms MreB - chaperonin complex formation

**Figure 3:**
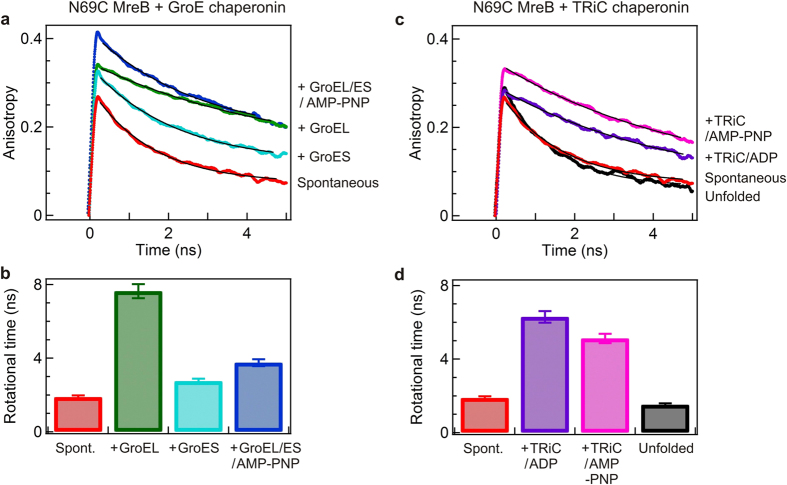
Time resolved anisotropy decay curves of Atto647 labelled N69CMreB. Representative time resolved anisotropy decay traces of the N69CMreB variant during GroE chaperone mediated refolding (**a)**, and in comparison with TRiC mediated refolding (**c**). Denatured N69CMreB diluted into 4 M GuHCl (*black*), refolding buffer (*red*), bound to GroEL (*green*), GroES (*cyan*), GroEL/ES/AMP-PNP (*blue*), TRiC/ADP (*marroon*), and TRiC/AMP-PNP (*pink*) respectively. The time resolved anisotropy decay (r_τ_) curve is calculated from time resolved HH and HV polarized decay raw traces. An appropriate portion of r_τ_ is fitted to a single-exponential decay model without deconvolution. The calculated rotational diffusion times of Atto647 labeled N69CMreB in GroE chaperonin mediated refolding (**b**) in comparison with TRiC mediated refolding (**d**).

Also herein the same single-molecule setup was employed to readily avoid monitoring of protein aggregates, which may interfere in bulk measurements. Local environmental based structural modulation of MreB arising from interaction with the chaperonin components can be monitored by fluorescence anisotropy experiments at various positions in the MreB structure. Figure 3a,c shows Atto647 labelled N69CMreB time resolved anisotropy decay traces in the presence of GroE mediated and TRiC mediated refolding, respectively in comparison with spontaneous refolding. An appropriate portion of the data is fitted by considering the single-exponential decay model, and calculated rotational diffusion times are summarized in Fig. 3b,d.

During spontaneous refolding the committed N69CMreB monomer seems to have reached a native-like globule state with an average anisotropy value of about 0.25 with a rotational diffusion time of ~1.9 ns (Fig. 3a). The increase in fluorescence anisotropy values in a range of ~0.3 to 0.4 is a significant indication of N69CMreb binding to GroEL alone, GroES alone, and also in concert when compared to spontaneous refolding (Fig. 3a). First, the observed increase in anisotropy shows that N69CMreB in the presence of GroEL is able to form a very slowly tumbling ~850-kDa N69CMreB/GroEL complex with a rotational diffusional time of 7.6 ns. The formation of a ternary N69CMreB/GroEL/ES/AMP-PNP complex decreases the rotational diffusional time to 6.8 ns (Fig. 3b). Importantly, we noted a noticeable increase in the anisotropy from 0.25 to 0.3 with a ~2.8 ns rotational diffusion time in GroES mediated refolding when compared to spontaneous refolding anisotropy (Fig. 3a,b). Such an effect could not be seen with FCS (which is sensitive to the cubic root of the molar mass), yet it becomes noticeable with fluorescence anisotropy (which is linearly sensitive to the molar mass)^30^. Second, the anisotropy also increased to into a range of 0.3–0.35 when N69CMreB binds to the TRiC chaperonin by forming a slow tumbling on average ~950-kDa complexes (N69CMreB/TRiC/ADP and N69CMreB/TRiC/AMP-PNP) with rotational diffusion times of ~6.3 ns and 5.1 ns, respectively (Fig. 3d). Hence, we conclude that TRiC recognizes and binds the folding MreB as it is diluted from 4 M GuHCl to folding conditions similar to the GroE chaperonin.

To verify that the anisotropy decays and rotational diffusion times found in N69CMreB are representative and are not an artifact depending on the mutation site position, we have also measured the time resolved anisotropy decays in the two other MreB mutants E258CMreB and N69CE258CMreB. The results are summarized in supplementary Fig. S2, and fully confirm our analysis on N69CMreB. Altogether it is clear that MreB binds to GroEL alone, GroES alone and also TRiC alone independently. All these time resolved results are well in accordance with our earlier discussed FCS observations on the interactions of MreB with the eukaryotic chaperonin TRiC and the prokaryotic chaperonin GroE system.

### Homo-FRET probes the conformation of MreB upon binding to chaperonins

While the FCS and single mutant fluorescence anisotropy demonstrate MreB–chaperonin complex formation, these methods lack information about the influence of the GroE or TRiC chaperonins on the MreB conformation during the folding process. To recover information about the conformational changes of MreB, we probe the fluorescence anisotropy decrease in the double mutant as compared to the single mutant case, which is induced by homo-FRET between the fluorescent mutation sites (see the Methods section for details). The steady state anisotropy traces of all MreB variants in various chaperone mediated refolding reactions in comparison with spontaneous refolding are summarized in supplementary Fig. S3.

**Figure 4:**
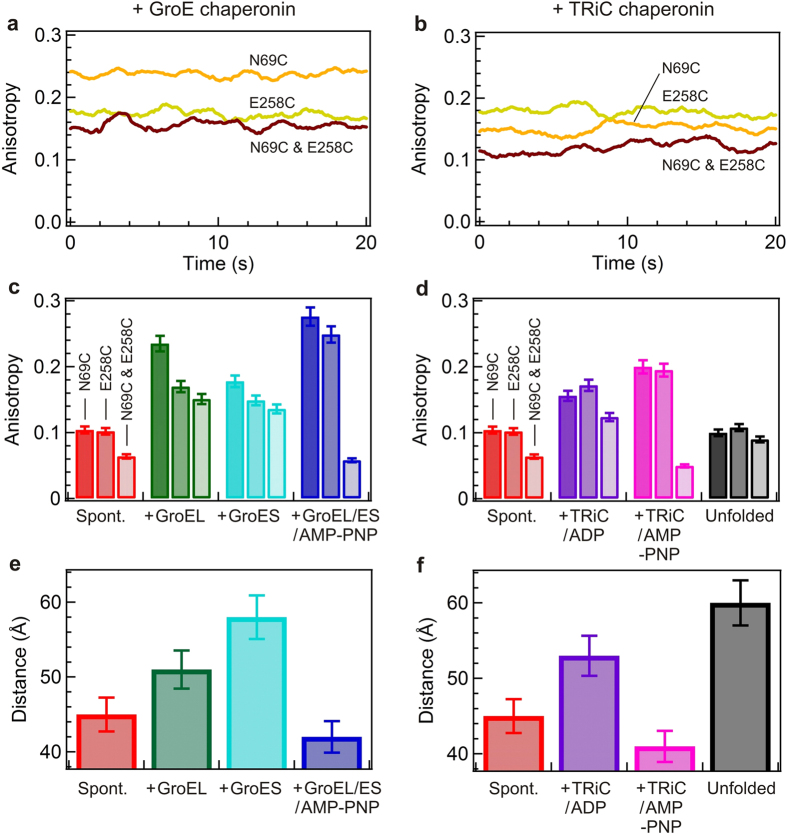
Homo-FRET reduces the anisotropy of labeled MreB variants. Steady-state anisotropy time trace of the denatured Atto647 labeled Mreb single variants N69CMreB (*orange*), E258CMreB (*green*) and their respective double variant N69CE258CMreB (*dark brown*), diluted into GroEL alone mediated refolding (**a**), in comparision with TRiC/ADP mediated refolding (**b**). Average anisotropy values of the Atto647-labeled MreB variants during refolding in the presence of the various chaperonins, in the presence of GroE chaperonin components (**c**) and in TRiC mediated refolding (**d**). In all cases N69CE258CMreB variant show lower anisotropies than their corresponding single variants due to the homo FRET. The GroE components and TRiC were present from the beginning before addition of the denatured MreB to the refolding buffer in the presence of GroEL alone (*green*), GroES alone (*cyan*), GroEL/ES/AMP-PNP (*blue*), TRiC/ADP (*maroon*), TRiC/AMP-PNP (*pink)* and in comparison with the spontaneous refolding (*red*) and unfolded state (*black*). The calculated FRET distances (in Ångström) from anisotropy in the presence of GroE chaperonin (**e**), *vs* TRiC chaperonin (**f**). The same color code is used as in (**c,d**) respectively.

Figure 4a,b show the reflected steady state anisotropy of the single mutants N69CMreB and E258CMreB and corresponding double mutant N69CE258CMreB in the presence of GroEL alone, and TRiC/ADP mediated refolding, respectively. The variations in the observed anisotropies in both of the single mutants N69CMreB and E258CMreB confirm that distinct GroEL/MreB, GroES/MreB, and TRiC/MreB complexes are formed. For the double mutant N69CE258CMreB, the fluorescence anisotropy values are lower due to the homo-FRET between the two fluorophores. This evolution and the occurrence of homo-FRET is confirmed for all the different complexes under investigation (Fig. 4c,d).

We compute the distance R between the N69C and E258C positions in MreB using the measured FRET efficiency deduced from the fluorescence anisotropy according to Eqs (4) and (5). The calculated distances between the fluorophores in the positions 69 and 258 under the different experimental conditions are presented in Fig. 4e,f. Theoretical distance between the 69 and 258 positions in MreB is ~20 Å (calculated by using the Yasara software, considering 1JCE pdb structure). However, the experimental distance reported is in a range of 41–45 Å for the final native-like MreB molecule. This discrepancy can be explained by considering that the insertion of fluorescent labels adds an extra distance due to the chemical linkers to the mutated amino-acids, typically of ~10 Å per label. Besides, we cannot rule out the hypothesis that mutations and insertions of fluorescent labels may prevent the target protein to fully reach its native state. While comparing the distances in Fig. 4e,f, it is noted that the distance significantly increases upon formation of GroEL/MreB, TRiC/ADP/MreB complexes in a range of 51–54 Å when compared to the spontaneous refolding, which shows a distance of ~45 Å.

### Comparison of the GroE and TRiC mechanisms of action

In this report the fluorescent probes in MreB are located in subdomains 2 (flexible floppy region) and 3 (nucleotide binding cleft region). Hence, the global modulation of the MreB structure can be monitored by calculating the distance between the 69 and 258 positions in MreB molecule. Our results in Fig. 4e,f show that the binding induced expansion of MreB occurs in both GroE and TRiC mediated refolding. As compared to the 45 Å distance in the MreB intermediate refolded spontaneously at 0.3 M GuHCl, the distance is increased by 6 Å in MreB bound to GroEL, and 9 Å in TRiC/ADP. Our results thus indicate that the bound non-native MreB is stretched less dramatically by GroEL as compared to TRiC/ADP. Moreover, the distance is remarkably decreased ~9 Å and ~13 Å upon addition of AMP-PNP to the GroE and TRiC system, respectively.

Our present results imply that non-native MreB is not stretched to the same extent when it binds to the GroE or the TRiC chaperonin systems. The differences in TRiC-bound and GroEL-bound MreB could, at least partly, be a consequence of differences in the apical domains of GroEL and TRiC. The GroEL apical domain recognizes the substrate protein through hydrophobic patches^31^,^32^,^33^, but the TRiC subunit CCTγ where the substrate interacts contains polar and charged amino acid residues^34^. In addition, the bigger size of the 90 Å TRiC internal cavity diameter^35^ as compared to 50 Å for GroEL^36^, may selectively expand distinct regions of the MreB molecule. All these observations stand in accordance with previous studies on the interactions of the MreB homolog β-actin with the eukaryotic chaperonin TRiC and the GroE system^13^,^21^.

Earlier studies from our group investigated the natural eukaryotic chaperonin system TRiC and its target protein β-actin^21^. The structural rearrangements occurring during the TRiC−β-actin interaction were probed using four distinct doubly fluorescein-labeled variants of β-actin. This rather detailed map revealed that TRiC has an active role in rearranging the bound β-actin molecule. The β-actin target was stretched as a consequence of binding to TRiC and was further rearranged in a second step as a consequence of ATP binding. Quantitatively, the initial stretching induced by TRiC on β-actin corresponds to a 12 Å increase of the distance between R39C and Q246C actin sites^21^. This value can be compared to the 4 Å distance increase found in the case of β-actin interacting with GroEL^13^. We observe a similar trend in our data reported herein, with a distance increase of 9 Å for MreB interacting with TRiC, and 6 Å for MreB interacting with GroEL. These results indicate that the active participation of the chaperonins in the folding process by forced unfolding appears to be evolutionary-conserved.

### Nucleotide hydrolysis during substrate release

Interestingly, addition of AMP-PNP to the GroES/EL/MreB or TRiC/ADP/MreB complex induces a significant compaction of the MreB substrate. The observed significant drop in anisotropy (Fig. 4c,d) and increased distance (Fig. 4e,f) between the probes in the double mutant N69CE258CMreB compared to the single mutants confirm a coherent pattern of structural modulation in MreB as a result of AMP-PMP nucleotide dependent re-arrangements of the chaperonin mediated refolding. The distance decreased dramatically by ~11 Å in the presence of AMP-PNP when compared to the absence of nucleotide for both GroEL and TRiC mediated refolding. In addition, the significant decrease in rotational time in the presence or absence of nucleotide AMP-PNP in both single mutants are very dramatic in both the GroE and TRiC systems when compared to its open state GroEL alone, or TRiC/ADP state, which confirms the earlier discussed local environmental effect.

### MreB stretching upon binding to GroES is most dramatic

A surprising result from previous homo-FRET experiments was the large distance increase of MreB induced by GroES binding to folding MreB comparable to or exceeding that of GroEL alone^28^ between positions 69 and 243. Herein between position 69 and 258 we observed up to 60 Å distance upon formation of the MreB-GroES complex as compared to the 45 Å distance for spontaneous refolding (Fig. 4e). Of significance, the MreB conformation in complex with GroES was even more stretched compared to GroEL or TRiC. As we already discussed, in the case of the small molar mass difference between the substrate and the chaperonin, measuring the anisotropy is more sensitive than FCS. The result that MreB binds to GroES is interesting since it has been suggested before that GroES does not interact with non-native proteins^37^. However, our results tend to indicate that GroES cooperates in substrate modulation during folding by significantly stretching MreB. Altogether the influence of GroES on MreB variants observed both at steady state and time resolved anisotropy measurements opens the prospect that GroES may acts as an unfoldase. Taken together with our previous observations that GroES also bound to carbonic anhydrase, serum albumin and lysozyme during refolding, which implicated recognition of a broad spectrum of protein substrates^28^, we suggest that GroES mechanism of individual substrate binding deserves further attention. It is very important herein to compare the structural state of MreB in the GroES-MreB complex with spontaneously refolded MreB and unfolded MreB. FCS data clearly showed that unfolded freely diffusing MreB occupies a large hydrodynamic radius (Fig. 2d), while MreB within the GroES-MreB complex appears globular with an apparent hydrodynamic radius less than half of unfolded MreB. The FRET data and the anisotropy hence in a similar comparison show that MreB is likely stretched out over the GroES structure (Fig. 4e,f) anchored most efficiently at position 69 (Fig. 4c,d) due to restriction of the probe (high anisotropy) in the single mutant.

**Figure 5:**
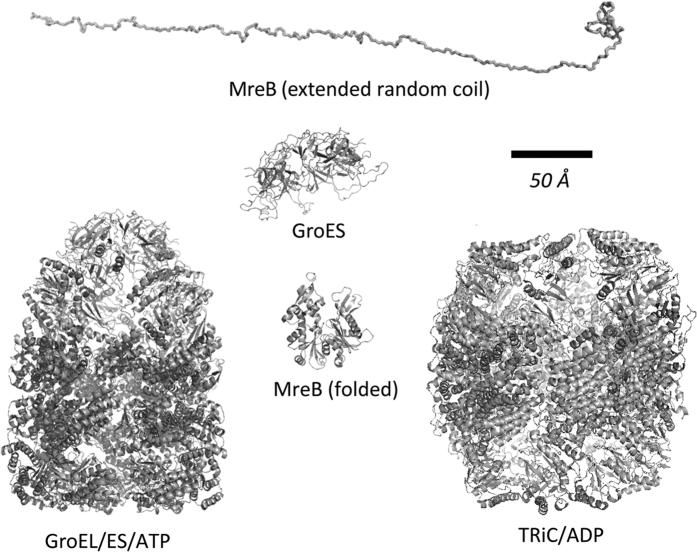
Molecular structures of MreB and chaperonins drawn to scale. The molecular structures of the proteins in the study illustrate the dynamic range of the employed techniques and the size dimensions attainable by the interactions between the chaperonins and MreB. The distance variations observed by FRET for various MreB conformations (Fig. 4e,f) are on the order of the diameter of the folded MreB molecule (50 Å) whereas the size of the concave binding cavity and distance between flexible loops of GroES are almost twice that size (90 Å). The conformational rearrangements imposed on the MreB molecule during chaperonin binding illustrate that the captured states are globular and not extended random coils. All structure models were generated by PyMol. Pdb codes used to make the figure: GroES and GroEL/ES/ATP (1AON)^36^, TRiC/ADP (4A13)^44^, MreB (1JCE). The extended random coil MreB was generated from 1JCE using the NMR server http://spin.niddk.nih.gov/bax/nmrserver.

An illustration of the molecular scales of GroES, GroEL/ES, TRiC, and MreB is shown in Fig. 5 to demonstrate the feasibility of our distance and anisotropy data. The distance variations we observe by FRET for the various MreB conformations (Fig. 4e,f) are on the order of the diameter of the folded MreB molecule (50 Å) whereas the size of the concave binding cavity and distance between flexible loops of GroES are almost twice that size (90 Å) (Fig. 5). To confirm that the effects caused by GroEL, GroES and TRiC are due to specific binding, we measured FCS and anisotropy for both single variants, during the spontaneous refolding reaction in the presence of 400 nM bovine serum albumin (Supplementary Fig. S4). No BSA-dependent effects on anisotropy were detected, well in agreement with our previous study on HCAII refolding by chaperonins in the presence of BSA^28^, supporting the conclusion that the effects observed for the respective chaperonin components were not caused by non-specific interactions, but rather by specific chaperonin component dependent interactions.

## Conclusion

In this study we investigated the refolding modulations of MreB, the prokaryotic homologue of actin upon interaction with type I and type II chaperonins. Both fluorescence correlation spectroscopy (FCS) and time-resolved fluorescence polarization anisotropy reported the binding of MreB to the chaperonin components GroEL, GroES and eukaryotic tail-less complex polypeptide 1 ring complex (TRiC) chaperonin. Fluorescence resonance energy transfer (FRET) measurements on site specific Atto labeled MreB variants were also used to quantify distance changes occurring upon chaperonin-assisted refolding. We report that TRiC alone, GroEL alone, and GroES alone influence the non-native MreB refolding individually and also in concert. The concerted interactions of GroEL and GroES are able to expand the initially collapsed MreB molten globule state and thereafter compress MreB. The GroEL-bound MreB molecule is somewhat more compact than TRiC/ADP-bound MreB. Importantly, our observations of the structural rearrangements induced by GroEL and TRiC show that similar stretching of the target protein is observed for both MreB and actin, indicating that the mechanism of the chaperonins is conserved during evolution. In addition the efficiency in stretching of the substrate during refolding by various chaperonins might be more distinctive and site-specific, which will provide the protein substrate with a new chance to fold correctly and avoid misfolding steps off the productive folding pathway. The current observations confirms the “binding induced expansion mechanism” occurring as an initial event, and also that the degree of stretching between subdomains of the non-native MreB molecule by GroEL alone, GroES alone and TRiC alone is unique. Encapsulation interactions are likely important to prevent aggregation during protein folding. Our data also implicates that the obligate need for GroEL + GroES to fold MreB^38^ may be a result of exceptional unfoldase activity of GroES on MreB to facilitate correct rearrangements of misfolded compact states. Such “binding induced unfolding” was previously reported necessary for β-actin chaperoned folding by TRiC^13^,^21^, and should be the subject of further studies. While our studies are limited to *in vitro* conditions using reporter-modified substrate protein, the implications for basic molecular biology of the cell would be obvious. Our data strongly supports that a revised mechanism for the rather passive role ascribed to GroES as a mere lid for GroEL would be needed. In contrast to such a passive role we propose that GroES can independently capture a folding substrate protein, stretch its conformation by binding induced unfolding and therafter deliver the substrate to GroEL for assisted folding. Our data appear valid under a range of protein concentrations; bulk measurements performed at micro molar concentrations^28^ as well as in sub micro molar conditions (here). While there is a possibility that the modified protein substrate (labeled MreB mutants) influences recognition of the chaperonins which prefer non-native conformations, the diversity of chaperonin induced conformations of MreB argues for specificity of the respective chaperonin component. Therefore, we made sure that the studies were made in parallel with TRiC, GroEL and GroES to allow such side-by-side comparisons. The most surprising finding of our study is the independent substrate binding and stretching activity of GroES. Importantly, we have previously discovered the independent binding of GroES to four different protein substrates including non-modified carbonic anhydrase, lysozyme and serum albumin. Notably, in that study the carbonic anhydrase core was also stretched by the GroES interaction, although in that case not to the same extent as by GroEL^28^. It is therefore evident that our data are valid and appear generic at least *in vitro*. If our results were to be viewed in the context of the the cell the molar concentrations of GroE components generally show an excess of GroES as compared to GroEL^39^,^40^. In a striking example Neidhardt and VanBogelen showed that under normal conditions (37 °C), the relative ratio of GroES versus GroEL was 1.9 and after heat shock it increased to 4.7. Quantification of chaperonin components in *E. coli* BM28 cells showed that these contained 20 μg of GroEL and 19 μg of GroES upon heat shock^41^. Taking the 6-fold difference in molecular weight into account proposes an 11.2-fold molar excess of GroES over GroEL which suggests that inside the cell there can be a large proportion of GroES available in a free form especially under stress. It has also been reported that from chromosomally expressed bacterial polycistronic groESL mRNA transcription and thus protein synthesis of GroES and GroEL are spatially organized according to gene order and due to restriction of ribosomal mobility this provides locally high concentrations^42^. Notably, GroES is encoded by a significantly shorter gene than GroEL, folds more rapidly and is synthesized first which should produce a pool of native GroES molecules being present as nascent GroEL is produced on the ribosome. Hence, interaction of GroES with the subsequently formed GroEL should be expected and makes it obvious to propose that GroEL is a substrate for GroES. Using similar arguments, for obligate substrate proteins by means of molar excesses of GroES, we propose that GroES captures the partially folded substrate protein and stretches it out over its interior cavity, the substrate protein is then peeled off from GroES by GroEL and is allowed to refold encapsulated in the cis-cavity. Subsequent folding and release in its folded conformation is mediated by ATP hydrolysis. This hypothesis also proposes that proteins too large to be encapsulated such as aconitase^11^ could be efficiently bound by excess GroES molecules rather peripheral from the GroEL apical domains. Subsequent GroEL assisted refolding can thereafter commence.

## Methods

### Expression and purification

GroEL and GroES and TRiC were expressed and purified as previously described^14^,^15^,^21^. Recombinant MreB was produced and purified as described by Moparthi *et al.*^28^. MreB variants were generated and purified using his-tag Ni–NTA fast flow column with 500 mM imidazole in PBS buffer at a flow rate of 1 mL/min, and the eluted fractions containing pure MreB variants as analyzed by SDS–PAGE were dialyzed against 1 mM NaH_2_PO_4_, 10 mM NaCl at 4 °C in 6-h intervals for 24 h.

### Labeling of MreB Cys mutants

The labeling of MreB by Atto647 was performed in the native state by incubating purified MreB in 0.1 M PBS buffer (pH 8.0) with a 10-fold excess of maleimide-Atto647 (Atto-tech) overnight at 4 °C. Cys modified MreB mutant proteins were labeled with the thiol-reactive maleimide derivative of the fluorophore Atto647 by following the protocol provided by the manufacturer (Atto-Tec). Removal of free Atto647 was performed by G25 Sephadex column (GE-Healthcare) and 8 h of dialysis. The concentration of the labelled MreB was calculated by using an extinction coefficient of 150,000 M^−1^ cm^−1^ at 647 nm. Sample purity and specificity were confirmed by matrix-assisted laser desorption/ionization mass spectrometry.

### Sample Preparation

Fluorescence measurements were performed on GroEL or GroES or TRiC containing samples with final concentrations of 40 nM Atto647 labelled MreB, 250 nM GroEL, 350 nM GroES, 250 nM TRiC, and 2.6 μM ADP or AMP-PNP in 0.1 M PBS, 10 mM KCl, 10 mM MgCl_2_, pH 7.5. In GroEL or TRiC mediating refolding samples a 1:1.6 molar ratio of GroEL or TRiC, and in GroES mediated refolding samples a 1:2.2 molar ratio of GroES to MreB were added to the refolding buffer. Atto647 labeled denatured MreB (in 4 M GuHCl) was mixed with unlabeled, denatured MreB in 1:3 molar ratios and was subsequently diluted into the GroEL or GroES or TRiC-containing samples and let the refolding reaction was led to proceed for 45 min at 30 °C. To make proper distance determinations on double mutant during refolding, and to avoid the risk of “inter-MreB homo-FRET” between Atto647 probes, which are located in different MreB molecules, has been minimized by mixing the labeled MreB with a 3-fold excess of unlabeled MreB before diluting the denatured protein into various buffers. In the case of GroEL/ES/AMP-PNP or TRiC/AMP-PNP mediated MreB refolding, AMP-PNP was added to the chaperonin mediated refolding buffer, and incubated for 15 min at 30 °C. Altogether in the case of GroEL/ES/AMP-PNP mediated MreB refolding, the sample contains in total of 160 nM (40 nM labeled, and 120 nM unlabled) MreB monomers, 3.5 μM GroEL monomers, 2.5 μM GroES monomers and 2.6 μM AMP-PNP respectively. Soluble aggregates were removed by centrifugation at 17,700 × g for 5 min. To avoid the viscosity effect of the solute in chaperonin mediated refolding, which normally gives rise to larger anisotropy values, we mixed denatured MreB with 0.1% Tween.

### Fluorescence Correlation Spectroscopy (FCS)

All FCS experiments were carried out at 22 °C on a custom-built confocal fluorescence microscope with a Zeiss C-Apochromat 40× 1.2NA water-immersion objective using 100 mM PBS, 10 mM KCl, 10 mM MgCl_2_ as a refolding buffer at pH 7.5. To diminish the surface interactions with the glass coverslip we added 0.1% Tween-20 (Sigma) to all the samples. The fluorescence intensity temporal fluctuations were analyzed with a hardware correlator (Flex02-12D/C correlator.com, Bridgewater NJ with 12.5 ns minimum channel width). All the experimental data were fitted using Eq. 1 by considering a single species and free Brownian 3D diffusion in the case of a Gaussian molecular detection efficiency:


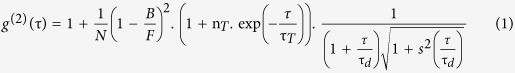


where N is the average number of molecules in the focal volume, F is the total fluorescence signal, B is the background noise, n_T_ is the amplitude of the dark state population, τ_T_ is the dark state blinking time, 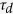 is the mean diffusion time and s is the ratio of transversal to axial dimensions of the analysis volume. The molecular diffusion coefficient, D and hydrodynamic radius, R_H_ were calculated previously described in detail^43^. We calibrate the parameter transversal waist in solvent viscosity, 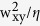 before each measurement on MreB samples by recording the FCS trace for Alexa Fluor 647 dyes which have a known hydrodynamic radius of 0.7 nm in pure water.

### Fluorescence anisotropy and homo-FRET of MreB mutants

Steady-state and time-resolved fluorescence anisotropy traces were recorded on the same FCS experiment setup after adding a polarizer before the fluorescence detection. The light excited by the 633 nm pulsed laser diode was horizontally polarized. The emission intensity was collected for two different polarization configurations, parallel (I_HH_), and perpendicular (I_HV_) to the excitation polarization. The anisotropy describes the difference in intensity of the emitted horizontal and vertical polarized light. The fluorescence anisotropy is calculated using the formula:


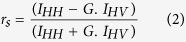


The difference in sensitivity of the detection system for horizontal and vertical polarized light is taken into account by the correction factor G = 1.3 in our setup. In the case of time-resolved anisotropy, the photodiode signal was recorded by a fast time-correlated single photon counting module (Picoquant GmbH) in time-tagged time-resolved (TTTR) mode. The overall temporal resolution of our system was 120 ps (full width at half maximum) which always remained very small as compared to the typical rotational time decay we measured. The fluorescence anisotropy decays were correctly interpolated by a mono exponential decay fit to determine the mean fluorescence rotational time τ_rot_ of different species. The goodness of the fit was assessed by the observation of randomly distributed residuals around zero and a χ^2^ value around 1.

The fluorescence resonance energy transfer (FRET) describes the distance-dependent energy exchange between electronically excited states from an excited donor to a ground state acceptor fluorophore. The energy transfer efficiency (E) is defined as:


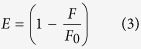


where F and F_0_ are the donor fluorescence intensities in the presence and absence of the acceptor, respectively. However in the case of homo-FRET the excitation and emission spectra are overlapping due to identical fluorescence probes. In such case the energy transfer can be determined from the decrease in the detected anisotropy of the fluorescent probes^21^. At steady state fluorescence anisotropy based energy transfer efficiency is calculated using the formula:


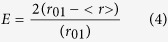


where r_01_ is the anisotropy in the absence of homo-FRET obtained from the average r_s_ value for the two corresponding single variants, and <r> is the observed steady-state anisotropy for the double mutant which is affected by the homo-FRET effect. The use of the average anisotropy (r_01_) from both single MreB variants to determine the distance avoids the uncertainties linked to the specific probe position in a single variant.

The measurement of the energy transfer efficiency enables computing the distance R between the fluorophores according to the formula:


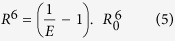


where R_0_ is the Förster radius, i.e. the distance between the flurophores for 50% energy transfer. The Förster radius for the Atto647 is calculated to be 51 Å. The distances were calculated from homo-FRET efficiency measurements between probes, and such transfer can be detected by changes in the anisotropy measurements of the single and double MreB variants.

## Additional Information

**How to cite this article**: Moparthi, S. B. *et al.* Differential conformational modulations of MreB folding upon interactions with GroEL/ES and TRiC chaperonin components. *Sci. Rep.* **6**, 28386; doi: 10.1038/srep28386 (2016).

## Supplementary Material

Supplementary Information
