# Supplementary material for: Differential conformational modulations of MreB folding upon interactions with GroEL/ES and TRiC chaperonin components
Source: Sci Rep. 2016 Jun 22;6:28386. doi: 10.1038/srep28386 (PMC4916439; doi:10.1038/srep28386)
Supplement: Supplementary Information [file srep28386-s1.pdf]

Differential conformational modulations of MreB folding upon interactions with GroEL/ES and TRiC chaperonin components

Satish Babu Moparthi[1], Uno Carlsson[2], Renaud Vincentelli[3], Bengt-Harald Jonsson[2], Per Hammarström[2], & Jérôme Wenger[1]

[1]CNRS, Aix Marseille Université, Centrale Marseille, Institut Fresnel, 13013 Marseille, France.

[2]IFM, Department of Chemistry, Linköping University, 581 83 Linköping, Sweden.

[3]Architecture et Fonction des Macromolécules Biologiques (A.F.M.B), UMR7257 CNRS, Université Aix-Marseille, Case 932, 163 Avenue de Luminy, 13288 Marseille Cedex 9, France.

Correspondence and requests for materials should be addressed to S.B.M (email: satish.moparthi@fresnel.fr)

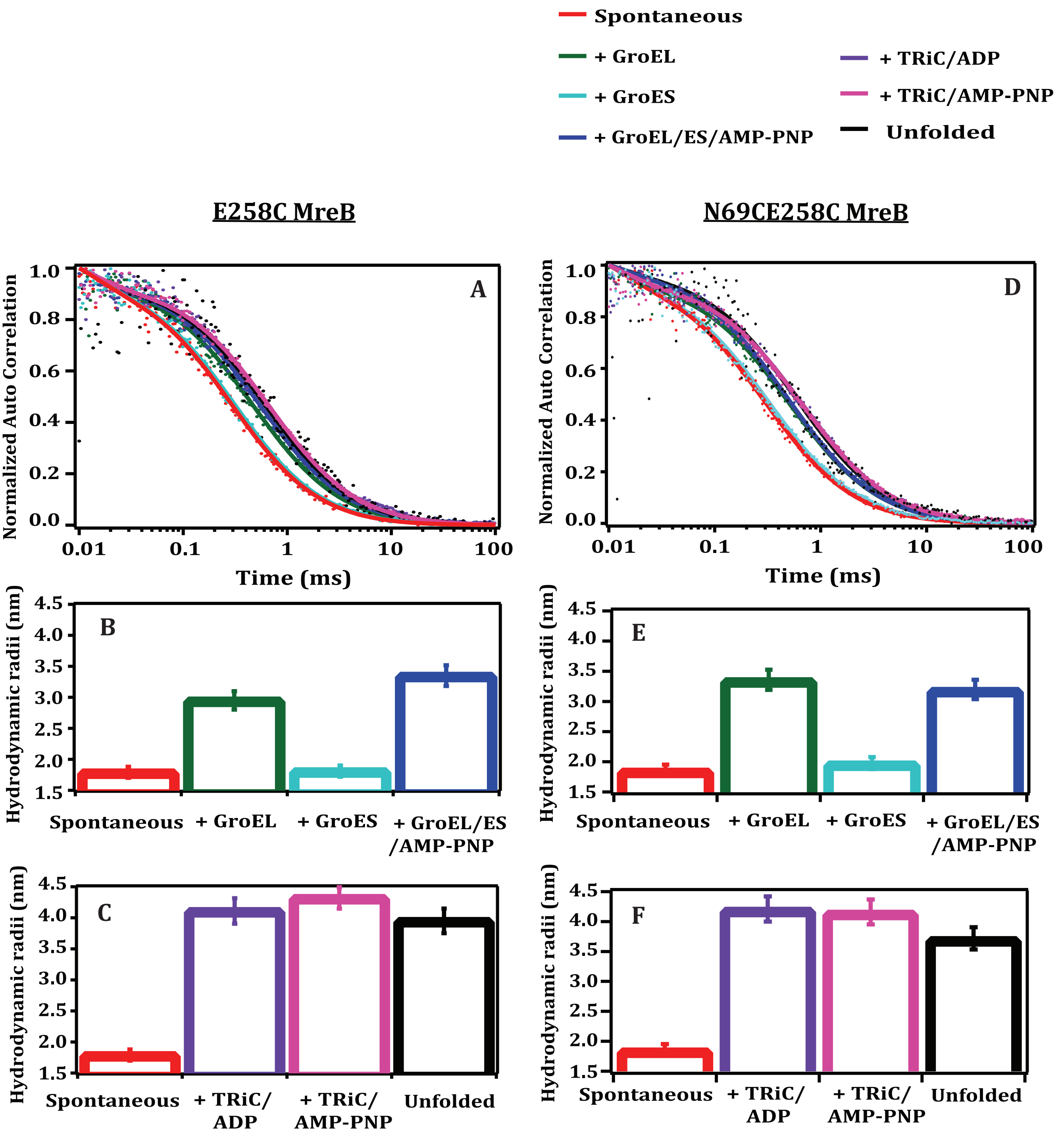

Supplementary Figure S1. FCS traces and Hydrodynamic radius of E258CMreB and N69CE258CMreb with various chaperons. A representation of normalized FCS correlation traces (A) and reflected hydrodynamic radius in the presence of GroE chaperone (B) and TRiC chaperone (C) of the Atto647 labelled E258CMreB variant. Normalized FCS correlation traces (D) and corresponding modulated hydrodynamic radius by GroE mediated refolding (E) and TRiC mediated refolding (F) of the Atto647 labelled N69CE258CMreB. The chaperonin components are present from the beginning before addition of the denatured MreB to refolding buffer in the presence of GroEL alone (green), GroES alone (cyan), GroEL/ES/AMP-PNP (blue), TRiC/ADP (maroon), TRiC/AMP-PNP (pink) and in comparison with the spontaneous refolding (red).

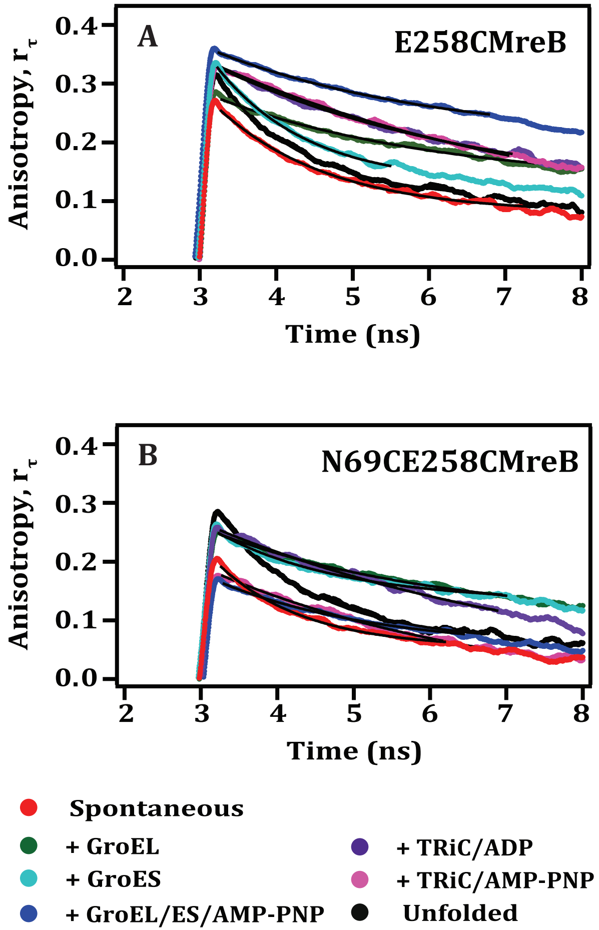

Supplementary Figure S2. Time resolved anisotropy decay curves of Atto647 labelled E258CMreb and N69CE258CMreB. Time resolved anisotropy decay traces of E258CMreB variant (A) and N69CE258CMreB (B) in the presence of various chaperone mediated refolding in comparison with spontaneous refolding (C). Denatured N69CMreB (black), diluted into refolding buffer (red), bound to GroEL (green), GroES (cyan), GroEL/ES/AMP-PNP (blue), TRiC/ADP (marroon), and TRiC/AMP-PNP (pink) respectively. The time resolved anisotropy decay (rτ) curve is calculated from time resolved HH and HV polarized decay raw traces, and also by considering G = 1.3.

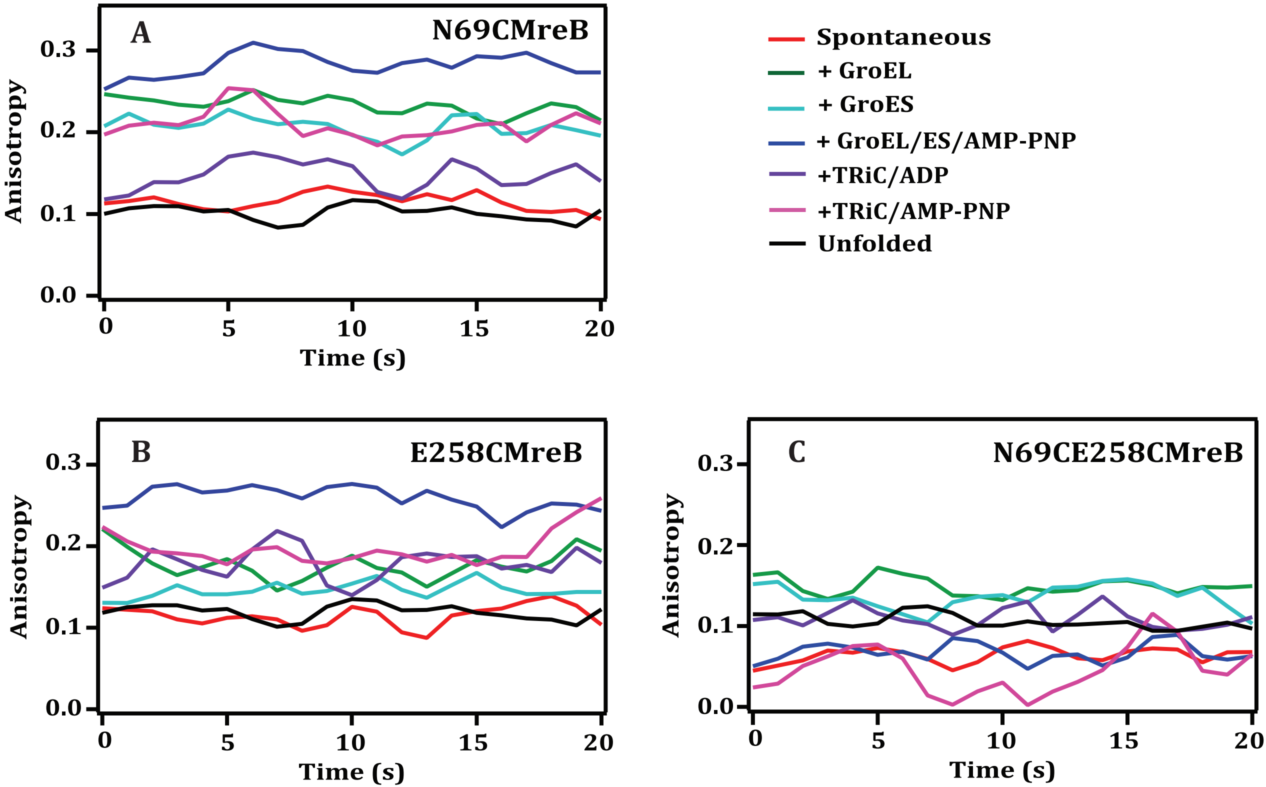

Supplementary Figure S3. Steady-state anisotropy time trace of labeled E258CMreB and N69CE258C variants. Steady-state anisotropy spectra of the denatured Atto647 labeled Mreb single variants N69CMreB (A), E258CMreB (B) and their respective double variantN69CE258CMreB (C), diluted into various chaperon conditions bound to GroEL alone (green), GroES alone (cyan), GroEL/ES/AMP-PNP (blue), TRiC/ADP (maroon), TRiC/AMP-PNP (pink) and in comparison with the spontaneous refolding (red) and unfolded state (black). In all cases N69CE258CMreB variant show lower anisotropies than their corresponding single variants due to the homo FRET.

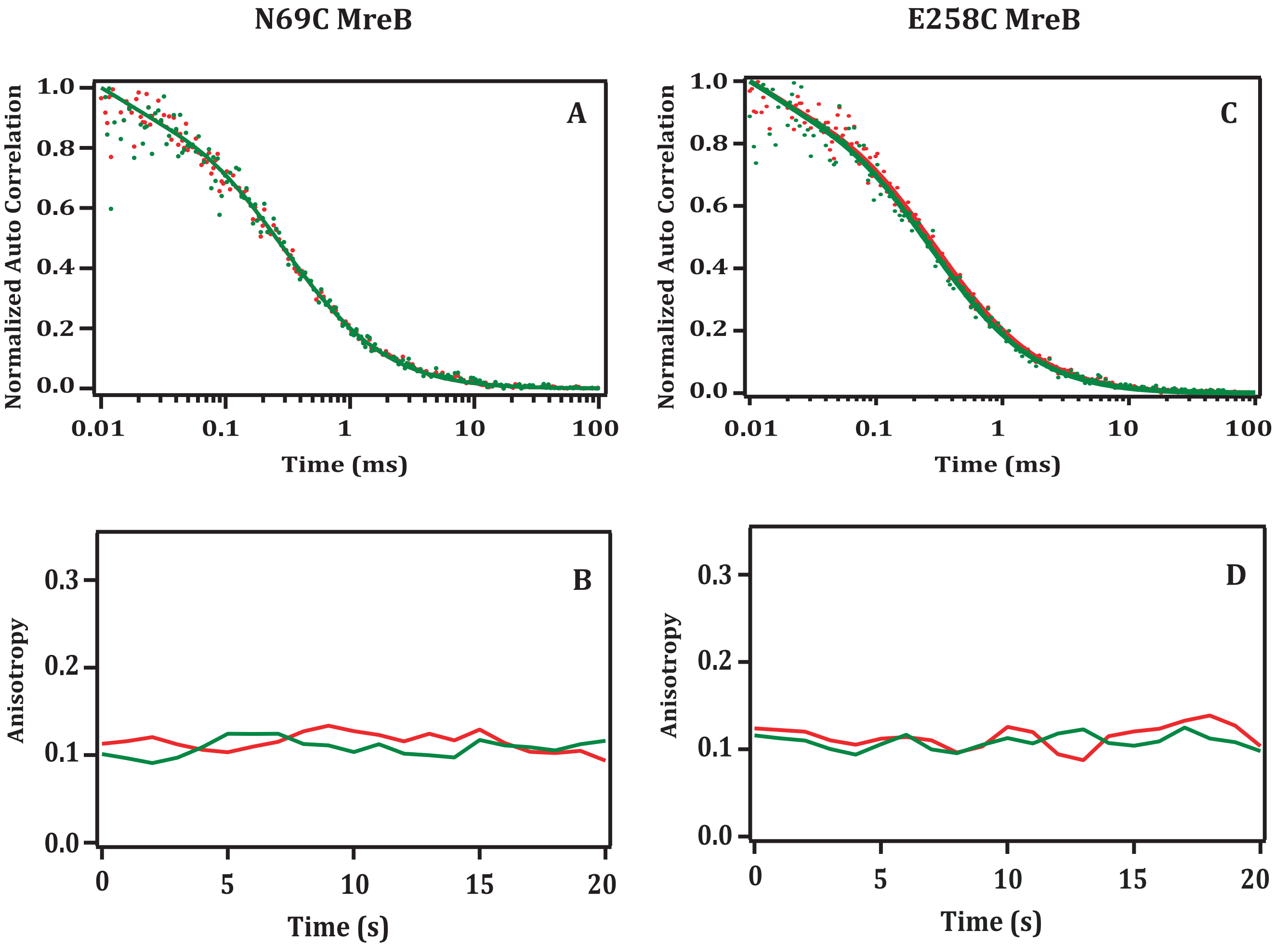

Supplementary Figure S4. FCS traces and Steady-state anisotropy time trace of labeled N69CMreB and E258C variants with BSA. A representation of normalized FCS correlation traces (A) and reflected anisotropy (B) of the Atto647 labelled N69CMreB variant in the presence of BSA . Normalized FCS correlation traces (C) and steady state anisotropy (D) of the Atto647 labelled E258CMreB in the presence of BSA. The spontaneous refolding buffer in the presence of BSA (green) in comparison with the spontaneous refolding (red).

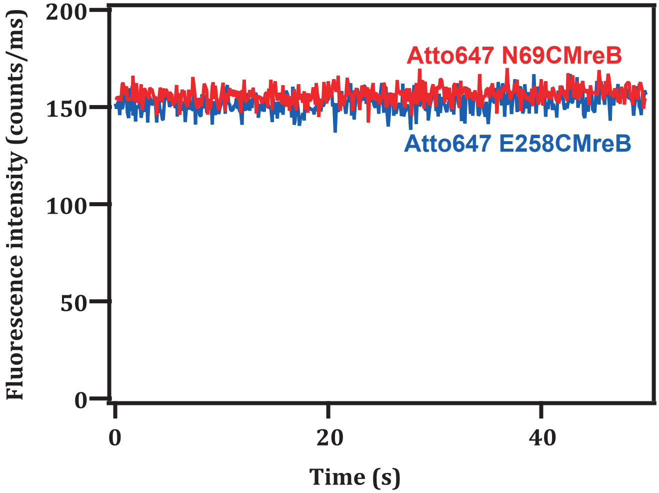

Supplementary Figure S5. Fluorescence intensity time trace of Atto647 labeled N69CMreB (red) and E258CMreB (blue).
